# ‘They ask no questions and pass no criticism’: A mixed-methods study exploring pet ownership in autism

**DOI:** 10.1007/s10803-022-05622-y

**Published:** 2022-06-09

**Authors:** Gray Atherton, Emma Edisbury, Andrea Piovesan, Liam Cross

**Affiliations:** 1 Department of Psychology, Edge Hill University, L39 4QP Liverpool, UK; 2 Università Iuav di Venezia, 30135 Venezia, VE Italy

**Keywords:** Autism, Pets, Anthropomorphism, Animals, Mental health, Quality of life, Mixed methods

## Abstract

Many autistic people cite a strong attachment to animals, and some studies suggest they may even show a bias towards animals over people. This mixed-methods study explored companion animal attachment in the adult autistic community. In a quantitative study with 735 people, we found that autistic adults were equally attached to their pets as neurotypicals but were less likely to own them, even though pet ownership corresponded with better mental health outcomes. Substituting pets for people also served as a compensatory mechanism for social contact in the autistic sample. In a second qualitative study, we explored the lived experiences of 16 autistic pet owners. The interpretive phenomenological analysis highlighted the benefits and the barriers to animal companionship. Together these mixed methods findings underline how pets improve the lives of their autistic owners. We conclude with specific recommendations for increasing animal companionship opportunities for autistic adults.

## Introduction

Some authors theorise that the socio-communicative differences often observed in autistic people stem from a lowered social motivation to engage in reciprocal interactions (Chevallier et al., 2012). This theory is grounded on research showing that autistic people exhibit reduced eye contact (Tanaka & Sung, 2016), mimicry (Eigsti, 2013), and reciprocal communication (Ochi et al., 2019), which negatively affects how others perceive them (Griffin, 2019; Grossman, 2015). However, others suggest that autistic people may only *appear* socially uninterested (Jaswal & Akhtar, 2019). Self-report data suggests autistic people have a strong interest in developing romantic attachments and friendships, though autistic people’s relationships may differ in style from neurotypical individuals (NTs) (Sedgewick et al., 2019; Teague et al., 2017). In short, while autistic people share the same social needs as NTs, differences in emotional experiences (Bird & Cook, 2013), social style (Sasson et al., 2017), and social competencies (Espelöer et al., 2021) may result in lost opportunities for social engagement.

Researchers are beginning to explore how autistic people use alternative means to fulfil specific social needs (Livingston & Happé, 2017). For example, they might bond with animals to fulfil their social engagement needs, and animals may even improve social cognition in autistic people. For instance, although autistic people are less responsive than NTs towards human stimuli (Chaminade et al., 2015a), other studies have shown that these difficulties are less pronounced, absent, or even reversed when interacting with animal stimuli. For example, Atherton & Cross (2019) showed that while those high in autistic traits perform worse on the faux pas test of theory of mind involving analysing social interactions, this pattern reverses if the test is given with an animal rather than human context. Valiyamattam et al., (2020) found that animal rather than human faces led to direct gaze and increased attention to eye regions in autistic children. Cross et al., (2019) also showed that a group of nonspeaking autistic adolescents were better at detecting emotions from the Karolinska directed emotional faces test when the faces were presented with lion and gorilla filters. Whyte et al., (2016) found hypoactivation in affective regions of the brain when autistic adolescents viewed human faces, while activation was typical when viewing animal faces. Finally, Prothmann et al., (2009) found that autistic children were more than twice as likely to interact with a dog than a human in a naturalistic setting, which was more than 16 times the amount of time they spent interacting with an object, underscoring the particularly social aspects of animal interaction.

Many studies that have found intact animal-human responsiveness in autistic samples have mainly used animal stimuli as a secondary control measure to study how autistic people perceive human social agents. However, looking at autistic people’s social responsiveness towards animals is in and of itself an important area of investigation. It introduces theoretical nuance to the more simplistic accounts of autistic people as mindblind or lacking empathy. Animals have complex social behaviours and emotional expressions that are processed and responded to similar to human stimuli (Desmet et al., 2017; Konok et al., 2015; Kujala et al., 2017). For instance, humans use ‘motherese’ to speak to pets, just as they would an infant (Prato-Previde et al., 2006), and they display similar distress responses to an animal cry as they do a human cry (Pongrácz et al., 2005). Similarities in the processing of human and animal social signals are unsurprising, given that humans and domestic animals co-evolved (Herbeck et al., 2017).

Given the similarities between human and non-human empathic displays, highlighting the intact human-animal bond in the autistic population is a stark rebuttal to the ‘pathos’ of autism (Duffy & Dorner, 2011). In essence, it contradicts the dehumanising narrative that autistic people are less loving, compassionate and invested in the emotions of themselves and others due to their social differences. Second, understanding the beneficial effect of animal-autistic people relationships is of practical importance. There is a paucity of cost-effective, timely interventions that support the mental health needs of autistic people (Gerhardt & Lainer, 2011).

Research on animal-assisted interventions that has been conducted on autistic child populations show promising results (for a review, see O’Haire 2013). For instance, the presence of animals reduced arousal and cortisol levels in autistic children (O’Haire et al., 2015; Viau et al., 2010) and promoted social responsiveness during interaction with NT children (O’Haire et al., 2015). When animals were used in conjunction with traditional therapy, autistic children were more socially responsive and experienced greater significant therapeutic gains (Gabriels et al., 2015; Grigore & Rusu, 2014; Martin & Farnum, 2002; Sams et al., 2006). Unsurprisingly, researchers have found that parents of autistic children commonly seek out animal-based interventions, with one-quarter of autistic children participating in animal-assisted therapy at some point in childhood and two-thirds of parents reporting improvements following the program (Christon et al., 2010).

While these results are encouraging, research on how the daily interaction and responsibility accompanying pet ownership is similar/different in autistic versus NT samples is equally essential as consistent animal companionship would arguably lead to more sustained gains. One sugy suggested that families with an autistic child may have increased rates of pet ownership (Carlisle, 2014) and, with the parents reporting that their children had formed a strong attachment to their pets (Carlisle, 2015). However, as is the case with much autism research, investigations into the effects of animal contact have focused almost entirely on child populations. It is equally essential to understand adult animal attachment given the benefits of animal contact and the underserved needs of the autistic adult community.

The present study employed a mixed-methods design with two overlapping aims to inform our understanding of the human-animal bond in autistic populations as compared to NTs. Study 1 used a quantitative methodology to explore whether autistic people and NTs differ in the rate of pet ownership, pet type and pet bond, and how the pet bond is linked to mental health. Study 2 employed a qualitative qualitative methodology (i.e., interviews) to investigate how pets make a difference in their autistic owners’ lives and how the animal-human bond may complement autistic people’s social needs.

## Study 1

### Method

#### Participants

A total of 326 individuals diagnosed with autism (ASC group) and 409 individuals who reported as neurotypical (NT group) completed the online survey. The ASC group were aged between 18 and 63 with a mean age of 28.64 (SD = 9.57) and included 176 males and 150 females. The NT group were aged between 18 and 79 with a mean age of 33.91 (SD = 13.05) and included 123 males and 286 females. Participants were recruited through social media channels (Facebook, Twitter and Reddit), autism websites and Prolific (Oxford, UK), an online research recruitment portal. Both targeted spaces for autistic individuals and more general online spaces were solicited. Specifically, we recruited individuals who were autistic and neurotypical, including individuals with and without pets. An autism diagnosis was confirmed by asking each participant whether they had an autism diagnosis, whether it was given by a medical professional, and the year it was given. This is similar to the method outlined by Baron-Cohen et al., (2015), which has been shown to correlate with the rate of an actual autism diagnosis. All participants were paid in line with UK minimum wage, and the survey took 30 min to complete. This study received ethical approval from Edge Hill University’s ethical review board and was conducted online via Qualtrics.

## Materials

All participants were asked to complete the following questionnaires, in the following order:

The Liebowitz Social Anxiety Scale (Liebowitz, 1987) is a 24-item scale measuring social anxiety related to fears and avoidance behaviours (e.g. telephoning in public, meeting strangers). Each item was responded to on two separate 4-point Likert Scales (Fear none - severe, Avoidance never - always).

The Satisfaction with Life Scale (Diener et al., 1985) is a 5-item scale measuring individuals’ satisfaction with life, (e.g. In most ways my life is close to ideal). This was responded to on a 7-point Likert Scale (Strongly Agree - Strongly Disagree).

The UCLA Loneliness Scale (Russell et al., 1980) is a 20-item scale measuring perceived loneliness, (e.g. I have nobody to talk to; there is no one I can turn to). This was responded to on a 7-point Likert Scale (I never feel this way - I always feel this way).

The Multidimensional Scale of Perceived Social Support (MSPSS) (Zimet et al., 1988), is a 12-item scale that measures perceived social support. (e.g. My family really tries to help me). This was responded to on a 7-point Likert Scale (Very Strongly Agree – Very Strongly Disagree).

The Autism Quotient (Baron-Cohen et al., 2001) is a 50-item scale measuring autistic traits and behaviours, (e.g. I enjoy social chit-chat,). This was responded to on a 4-point Likert Scale (Definitely Agree – Definitely Disagree).

Pet owners only (265 ASC and 374 NT) were also asked to complete the following questionnaires:

The Lexington Attachment to Pets Scale (LAPS) (Ramírez et al., 2014) is a 23-item scale used often to measure individuals’ attachment to pets (e.g., I believe my pet is my best friend). This is taken on a 4-point Likert scale (I strongly disagree - I strongly agree). The questionnaire is divided into 3 subscales: general attachment (score range: 0–33), people substitution (score range: 0–21), animal rights (score range: 0–15).

The Critical Pet Rating (Epley et al., 2008) is a 14-item scale which measures the degree to which individuals anthropomorphise their pets by having people rank human and non-human characteristics and how they apply to pets, (e.g., rate how creative (anthropomorphic trait) your pet is on a scale of 0 to 100; how agile (non-anthropomorphic trait) from 0 to 100). The anthropomorphic traits are further divided into two subscales, the pet rating social, pet rating non-social. Pet rating social items include the adjectives thoughtful, considerate, and sympathetic, which relate to social connection (score range: 0-300). The non-social anthropomorphic adjectives embarrassable, creative, devious, and jealous were also assessed (score range: 0-400).

When completing the Critical Pet Rating questionnaire, pet owners were requested to indicate which type of pet they thought about while completing the questionnaire. Participants could have indicated dog, cat, aquatic animal, bird, small pet (e.g., mammal and rodent), herptile, or outdoor pet (e.g., horse, goat, etc.). This was used in the below analyses to divide participants based on the pet type they owned. However, due to low numbers, participants who indicated anything except dog and cat were grouped together, resulting in 3 pet types: dog, cat and other.

Finally, all post-hoc analyses reported below were Bonferroni corrected.

The study variables were grouped in two clusters to improve readability: General Attachments, People Substitution, Animal Rights, Pet Rating Social, and Pet Rating Non-Social, and are referred to as pet relationship variables. In contrast, Performance Anxiety, Social Anxiety, Performance Avoidance, Social Avoidance, Life Satisfaction, and Loneliness are referred to as Mental Health variables.

## Results

Table 1 shows the means and ranges of the study variables divided by the two groups (ASC and NT). All variables were not normally distributed (all *p*s < 0.001; Kolmogorov-Smirnov tests reported in Supplementary Materials). The variables were highly correlated with the other variables of the same cluster (all *p*s < 0.001; Spearman’s correlations reported in Supplementary Materials).

**Table 1:** Means (with minimum and maximum scores) of the study variables were divided between individuals with and without a diagnosis of autism

*Cluster*	*Variable*	*NT*	*ASC*
Pet Relationship	General Attachment	37.62 (11–44)	37.40 (13–44)
	People Substitution	20.77 (7–28)	21.49 (7–28)
	Animal Rights	17.09 (5–20)	17.03 (5–20)
	Pet Rating Social	166.03 (0-300)	164.56 (0-300)
	Pet Rating Non-Social	147.17 (0-378)	154.30 (0-392)
Mental Health	Performance Anxiety	16.4 (0–39)	21.90 (0–39)
	Social Anxiety	15.00 (0–33)	21.31 (0–33)
	Performance Avoidance	12.56 (0–39)	18.65 (0–39)
	Social Avoidance	12.06 (0–33)	18.88 (0–33)
	Life Satisfaction	21.33 (5–35)	17.08 (5–35)
	Loneliness	24.35 (0–59)	34.96 (0–60)

### Pet ownership and pet type distribution between ASC and NT

A Pearson’s Chi-square was conducted to test whether people diagnosed with autism were more likely to own, or have owned, a pet during their life. There was a significant association between pet ownership and diagnosis (*χ*^2^(1) = 16.47, *p* < .001). As highlighted in Table 2 those with ASC were less likely to have owned a pet during their life than NTs.

**Table 2:** Number (and percentage) of pet owners and non-pet owners as a function of diagnosis

*Group*	*Pet Owners*	*Non-Pet Owners*
*ASC*	265 (81.3%)	61 (18.7%)
*NT*	374 (91.4%)	35 (8.6%)

A Pearson’s Chi-square was then conducted to test whether there was an association between diagnosis group and the type of pet owned. Only pet owners were therefore included in the Chi-square analysis. There was a significant association between diagnosis group and pet type (*χ*^2^(2) = 7.23, *p* = .03). Table 3 suggests that, although dogs were the most common pet for both ASC and NT groups, those with ASC were more likely to own cats and other pets than NTs.

**Table 3:** Number (and percentage) of dog, cat and other pet owners as a function of diagnosis

*Group*	*Pet type*		
	*Dog*	*Cat*	*Other*
*ASC*	128 (48.3%)	98 (37.0%)	39 (14.7%)
*NT*	220 (59.0%)	112 (30.0%)	41 (11.0%)

### Pet ownership and diagnosis effects on quality of life

To test whether the quality of life differed between pet owners and non-pet owners and across diagnosis groups, a MANOVA was conducted with pet ownership (owners and non-owners) and diagnosis group (ASC and NT) as between-subject factors and the six Mental Health variables as the dependant variables.

The MANOVA showed no significant interaction effect of diagnosis and pet ownership on the Mental Health variables (Pillai’s Trace = 0.01, *F*(6, 726) = 0.78, *p* = .58, *η*_*p*_^*2*^ = 0.01). There was however a significant main effect of diagnosis group on the Mental Health variables (Pillai’s Trace = 0.08, *F*(6, 726) = 11.15, *p* < .001, *η*_*p*_^*2*^ = 0.08). Namely, there was a significant main effect of diagnosis group on all Mental Health variables (all *p*s < 0.001). Post-hoc tests indicated that those with ASC had higher scores for Performance and Social Anxiety, Performance and Social Avoidance and Loneliness compared to NTs, meanwhile NTs had higher scores for Life Satisfaction compared to those with ASC (all *p*s < 0.001). There was also a significant main effect of pet ownership on the Mental Health variables (Pillai’s Trace = 0.02, *F*(6, 726) = 2.92, *p* = .022, *η*_*p*_^*2*^ = 0.02). However, this was limited to Life Satisfaction only (*F*(1, 731) = 11.79, *p* = .001, *η*_*p*_^*2*^ = 0.02). Post-hoc tests indicated that pet owners had higher scores for Life Satisfaction compared to non-pet owners (*p* = .001).

In summary, NTs reported higher Mental Health scores, as indexed by all the included measures, compared to those with ASC. Furthermore, pet owners reported greater life satisfaction than non-pet owners. This was true for both the NT and ASC groups.

### Pet relationship for ASC and NT

A series of Mann-Whitney U tests were conducted to assess whether ASC and NT participants had a similar relationship to pets. Analyses indicated that ASC and NT groups scored similarly for General Attachment (U(Nasc = 257, Nnt = 365) = 45,770, *p* = .61), Animal Rights (U(Nasc = 262, Nnt = 367) = 47,877, *p* = .93), Pet Rating Social (U(Nasc = 264, Nnt = 374) = 48,553, *p* = .72) and Pet Rating Non-Social (U(Nasc = 264, Nnt = 374) = 47,286, *p* = .36). However, ASC participants scored higher for People Substitution compared to NTs (U(Nasc = 264, Nnt = 368) = 43,534, *p* = .03), suggesting that ASC individuals were more likely to use their pet as a substitute for human companionship compared to NTs.

### Relationship between pet relationship and quality of life

Tables 6 and 7 report Spearman’s correlations between the pet relationship variables and the Mental Health variables separated by diagnosis group. Some correlations were significant for both groups: People Substitution was positively related to all Mental Health measures except Life Satisfaction (all *p*s < 0.05); Pet Rating Non-Social was positively related to Performance and Social Anxiety and Performance and Social Avoidance (all *p*s < 0.05); and Animal Rights was positively related to Performance and Social Anxiety (all *p*s < 0.05). However, two correlations were significant only for the NT group: Animal Rights and Pet Rating Non-Social were positively related to Loneliness (all *p*s < 0.05).

In contrast, and more interestingly, multiple correlations were significant only for the ASC group: General Attachment did not correlate with any of the Mental Health measures in the NT group. However, it was positively related to all Mental Health measures except Life Satisfaction in the ASC group (all *p*s < 0.05). Similarly, Pet Rating Social did not correlate with any of the Mental Health measures in the NT group. Meanwhile, it was positively related to Performance Anxiety and Loneliness in the ASC group (all *p*s < 0.05). Finally, Animal Rights was positively related to Performance and Social Avoidance (all *p*s < 0.001), and Pet Rating Non-Social was positively associated with Life Satisfaction (*p* < .05) in the ASC group only.

**Table 4:** *Spearman’s correlations between* Mental Health *measures and pet relationship measures within ASC participants*

	*General Attachment*	*People Substitution*	*Animal Rights*	*Pet Rating Social*	*Pet Rating Non-Social*
*Performance Anxiety*	0.22***	0.29***	0.15*	0.14*	0.21***
*Social Anxiety*	0.31***	0.35***	0.26***	0.11	0.15*
*Performance Avoidance*	0.25***	0.34***	0.21***	0.05	0.19**
*Social Avoidance*	0.32***	0.36***	0.29***	0.06	0.14*
*Life Satisfaction*	0.06	-0.03	0.01	0.09	0.14*
*Loneliness*	0.15*	0.26***	0.05	0.16**	0.06

*p < .05, **p < .01, ***p < .001

Note: significant correlations unique to the ASC group are underscored

**Table 5:** *Spearman’s correlations between* Mental Health *measures and pet relationship measures within NT participants*

	*General Attachment*	*People Substitution*	*Animal Rights*	*Pet Rating Social*	*Pet Rating Non-Social*
*Performance Anxiety*	0.06	0.18***	0.10*	0.05	0.17***
*Social Anxiety*	0.10	0.20***	0.12*	0.06	0.16**
*Performance Avoidance*	0.02	0.13*	0.03	0.02	0.11*
*Social Avoidance*	0.06	0.15**	0.06	-0.01	0.13*
*Life Satisfaction*	0.03	-0.09	-0.01	0.01	-0.01
*Loneliness*	0.05	0.21***	0.11*	0.05	0.15**

*p < .05, **p < .01, ***p < .001

Note: significant correlations unique to the NT group are underscored

### Mediation between diagnosis, social avoidance and people substitution

**Fig. 1:**
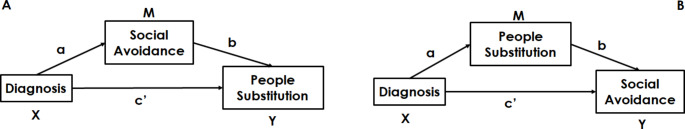
Mediation analyses models

The positive relationship between people substitution and social avoidance amongst those with ASC could suggest one of two different outcomes. Those with ASC were more likely to use their pet as a substitute for people than NTs because they were more socially avoidant, or those with ASC were more socially avoidant through the developed use of their pet as a substitute for people. We, therefore, tested two mediation analyses to assess whether social avoidance (M) mediated the effect of autism (X) on people substitution (Y) (Fig. 1, Model A), or whether people substitution (M) mediated the effect of autism (X) on social avoidance (Y) (Fig. 1, Model B).

Mediation analyses were conducted using PROCESS v3.5.3 (www.processmacro.org) developed by Hayes (2017). Specifically, Model 4 from the macros created for SPSS was used. Each mediation analysis first calculated the total effect of X on Y (*c*). Mediation analysis then calculated the effect of X on the mediator (*a*) and the effect of the mediator on Y (*b*). The indirect effect of X on Y via M (*ab*) was tested using a bootstrap estimation approach with 5000 samples. The direct effect of X on Y (*c’*) was also calculated. All coefficients have been reported in Table 6.

**Table 6:** Mediation coefficients [95% confidence intervals]

*Model*	*Effect of X on M (a)*	*Effect of M* *on Y (b)*	*Indirect effect (ab)*	*Direct effect (c’)*	*Total effect (c)*	*Degree of mediation*
A	-7.04* **[-8.23, -5.84]**	0.17* **[0.12, 0.21]**	-1.18* **[-1.56, -0.83]**	0.46 [-0.33, 1.25]	-0.72 [-1.47, 0.02]	Complete
B	-0.72 [-1.47, 0.02]	0.43* **[0.31, 0.55]**	-0.31 [-0.64, 0.01]	-6.72* **[-7.88, -5.57]**	-7.04* **[-8.23, -5.84]**	None

*p < .001

Complete mediation was obtained when the mediator was social avoidance (Model A); meanwhile, there was no mediation when the mediator was people substitution (Model B). In fact, although diagnosis group just failed to reach a significant total effect on people substitution (*p* = .058), the mediation analysis showed that autism had an indirect effect on people substitution via social avoidance. This supports the hypothesis that those with ASC were more likely to use pets as people substitutes because they were more socially avoidant. In other words, pets do not drive social avoidance, but are instead used as a compensatory mechanism to fulfil social needs in light of social avoidance.

### Pet type and diagnosis effects on quality of life

To test whether the quality of life of pet owners differed across pet types and diagnosis group, a MANOVA with pet type (dog, cat, other) and diagnosis group (ASC and NT) as between-subject factors and the six Mental Health variables was conducted.

The MANOVA showed no significant interaction effect of group and pet type on the Mental Health variables (Pillai’s Trace = 0.03, *F*(12, 1256) = 1.67, *p* = .07, *η*_*p*_^*2*^ = 0.02) and no significant main effect of pet type on the Mental Health variables (Pillai’s Trace = 0.02, *F*(12, 1256) = 1.02, *p* = .43, *η*_*p*_^*2*^ = 0.01). There was however a significant main effect of diagnosis group on the Mental Health variables (Pillai’s Trace = 0.15, *F*(6, 627) = 17.69, *p* < .001, *η*_*p*_^*2*^ = 0.15). Namely, there was a significant main effect of diagnosis group on all Mental Health variables (all *p*s < 0.001). Post-hoc indicated that those with ASC had higher scores for Performance and Social Anxiety, Performance and Social Avoidance and Loneliness compared to NTs, meanwhile NTs had higher scores for Life Satisfaction compared to those with ASC (all *p*s < 0.001).

In summary, within pet owners, we confirmed that NTs reported higher quality of life scores, as indexed by all the measures we included, compared to ASCs. Furthermore, there was no effect of pet type (i.e., dog, cat, or other) on quality of life for NT nor ASC participants.

## Study 1 discussion

In this first study, we compared ASCs and NTs regarding their bond with animals and how it was related to their general mental health. In summary, we found that, compared to NTs, autistic people were less likely to own pets overall, more likely to own non-dog pets, reported lower quality of life overall, but had higher life satisfaction if they owned a pet. They were also just as attached to their pets, and the type of pet they had did not alter these effects. Like NTs, autistic people were just as conscious of animal rights and likely to see animals as humans. However, they did differ in that they were more likely than NTs to substitute a pet for a person. The mediation analyses showed that this stemmed from social avoidance, meaning that outside pressures that led to autistic people having more insular lives resulted in them using their pets to channel their needs for contact. This finding is particularly significant because it underscores the importance of pet ownership to autistic people and highlights the preserved social motivations in this population. Finally, general pet attachment was not related to mental health variables in NTs. For the ASC group however, pet attachment was postively related to all mental health variables, with the exception of satisfaction with life.  

## Study 2

### Methods

#### Participants and design

To better understand the relationship between autistic adults and companion animals, individuals aged 18 years and over with a diagnosis of autism were recruited from Study 1 to participate in semi-structured interviews. Specifically, all participants who participated in Study 1 were asked at the end of the survey to email the research team if they met the criteria of (a) possessing a diagnosis of autism and (b) having ever owned a pet. The first eight males and eight females who emailed back were then contacted to schedule the interview. The recruitment process resulted in international participation from individuals in countries including the United Kingdom, Canada, Czech Republic, Sweden, Germany, and Denmark. A total of 16 autistic adults (mean age 34.75, range 18–63, 7 females, 7 males, 2 non binary/other, 11 Caucasian British, 1 each of Caucasian Swedish, Canadian, Czech, 1 Asian, and 1 Native American) participated in the semi-structured interviews.

An interpretative phenomenological analysis (IPA) framework (Smith and Shinebourne, 2012) was employed in Study 2. This method, which corresponds with the qualitative analytical approach used in this research, aims to unravel the meanings behind individual experiences central to this study’s purpose, in line with previous work on autistic lived experiences (Atherton et al., 2018). According to Howitt (2019), IPA assumes that individuals are the experts in their own lives. The process of IPA can gain insight into memories and lived experiences that remain significant to the participant (Smith and Shinebourne, 2012). Mutual misunderstanding between autistic and non-autistic people can create difficulties when trying to understand the experiences of autistic people. However, IPA offers greater flexibility as it seeks to establish an equality of voice between the researcher and participant. This in and of itself can aid in understanding how autistic people conceptualise their own lives (Howard, Katsos and Gibson, 2019).

## Procedure and materials

Interviews were conducted in a private setting to respect participant confidentiality using online communication platforms such as Skype or WhatsApp. A recording device was used to audio record the interviews, which were later transcribed using a secretarial/ playscript method. The interview protocol (See Appendix C) was designed and based on several measures which examined pet relationships and mental health from Study 1. The semi-structured interview protocol consisted of 45 questions, and the interviews lasted approximately 50 min. Example questions included “Can you tell me about a specific pet you have owned?” and “Can you describe any times when you might feel lonely?” However, the IPA design of this study allowed flexibility throughout the interviews, which encouraged participants to take the lead in telling their own stories. Ethical approval was granted by the Research Ethics Committee at Edge Hill University. Participants were given information that outlined the study’s details prior to the interviewing process. All participants granted consent, and they were all adults aged 18 years and over. Each participant was reminded of the nature of the project before interviewing commenced and was paid £15 for their participation.

## Results

Consistent with Graneheim and Lundman’s (2004) guidelines, pieces of text in the transcripts that the researchers considered as significant to the participant’s experience were highlighted. Meaningful information extracted from the data was then condensed into word codes and aggregated into themes (i.e. “compassion for animals” or “social challenges”). To avoid interviewer bias, two researchers independently coded and grouped themes for each transcript before conducting the second stage analysis, involving discussion between the researchers to consider their findings and agree on themes for each interview. The final stage of analysis involved the identification of master themes that were consistently present within the overall data (Griffith et al., 2012). This process unearthed four overarching master themes which were divided into subthemes (Smith & Shinebourne, 2012). All themes and subthemes can be found in Table 7.

**Table 7:** Frequency count of themes and subthemes within the data

Themes	Subthemes	Frequency
Pets with Benefits		16
	Physical benefits	11
	Mental benefits	16
	Tactile stimulation	9
	Meaning and Purpose	7
Pets As A Social Alternative		16
	Socialising in the animal world	4
	No judgement	7
	Anti-masking	10
	Social norms	10
	Practice and honing of skills	11
	Body language	12
Pets As A Social Lubricant		14
	Pets allow for social integration	12
	Pets allow for social control	6
	Connecting with other pet owners	9
Barriers And Breakthroughs To Pet Ownership		14
	Logistical barriers	8
	Mental barriers	5
	Taking the plunge	5
	Responsibility is good	10

Frequency of themes demonstrates how many interviews out of 16 were recorded as possessing this theme. Frequency of subtheme demonstrates the number of times this subtheme was recorded within the total data.

An additional method was used as part of the research procedure known as member checking to reduce the potential for researcher bias and ensure participants’ voices were captured. The member check process and final thematic aggregation goal was to identify themes consistently present in the data and central to the participants’ authentic experiences. This process involved returning qualitative analysed data to participants to check and confirm the results (Bartz et al., 2016). Eight of the 16 participants responded with feedback which was incorporated accordingly.

All person and pet names have been changed to maintain anonymity.

When providing interview extracts, the following textual conventions are used:

Words omitted to shorten quote: ….

Explanatory information provided by authors: [text]

### Theme 1. Pets with benefits

A recurring theme throughout the interviews was that pets provide owners with physical and mental health benefits. Some of this stemmed from the responsibility of owning a pet, and a need to care for them properly. Dog owners, for instance, were mindful of the need to exercise their pets, which in turn led them to partake in physical activities:

She gets me a little bit of exercise sometimes because I go out walking with her and sometimes like especially in lockdown at the start, I didn’t really want to leave the house at all … But having to walk a dog allows me to go out a little bit more. And now I go out quite a lot and I just go out for runs actually now and I also walk her.

For many, pets also took care of their owners. In line with the common understanding of the role of a support animal, pets allowed owners to calm down in moments of stress and emotional upheaval. Much of this stemmed from animals’ nonverbal support when participants felt overwhelmed in other relationships.

I could be borderline having a complete meltdown over something, but if I catch it at the right time, I can go sit down with Teddy or Jonah my other cat and just stay with them for a few minutes. And while it won’t get rid of it entirely, it will bring it to a controllable level.

Many discussed being able to use pets to discuss their problems, and this helped them form a clear response to an issue they were struggling with or an emotion they were trying to regulate:

I think the times that I was anxious when I was a teenager and I had an animal, it went away very quickly. And I was able to bounce that off them and almost have a conversation with them in my head. I focused on them rather than the problem.

A key aspect of the therapeutic benefit of pets was the tactile stimulation that pets provided their owners. Participants discussed how the weight of a pet, and the sensation of touching fur or even scales was soothing in and of itself.

I stop crying because she calms me down by stroking her. And then she usually licks my hands, and, I know it sounds gross, but it’s actually really satisfying. … [her tongue] has a nice texture to it. So, she basically licks my hands and my arms and then and then that calms me down, and then I stroke her at the same time. And then I stop crying, and then I’m okay.

Participants were aware of the aspects of their autistic traits that tend to lead them to invest more time in what they considered to be less productive activities, such as excessive engagement with computer or video gaming. Pets provided a bridge of sorts for engaging in a relationship that brought them pleasure but also kept them grounded and deterred them from what they felt were problematic behaviours. Explains one participant:

About 10 years ago I started playing computer games and I could feel myself getting addicted to them, to the point where I felt resentment for other things in life getting in the way. Now I think if I didn’t have those responsibilities of having the pets there as a distraction, something to spend time with, something to focus on, I would probably end up online even more.

Through a love of pets, many became involved in rescuing animals and working with animals in the community. Taking in animals and helping others with pet issues became a priority. It gave them a feeling of accomplishment and aligned with their value system of treating living creatures with kindness and love:

[Loving animals] led me to volunteer at a cat sanctuary. I volunteered there for seven years, which was the only thing that got me out of the house on the weekends and summer holidays and things … At the sanctuary they gave me a lot of responsibility and I really took that on, and I would be there as much as they would let me.

### Theme 2. Pets As A Social Alternative

The interviews were laced with stories of social difficulties that participants had experienced throughout their lives, such as social anxiety, rejection, and trauma, mainly stemming from being poorly understood by neurotypicals. Animals provided an alternative way to experience social interaction that was free from the stresses that came with human contact:

All the situations that make autists uncomfortable don’t exist with animals, they’re gone. No awkward conversation, no unpredictable events and it’s an easy environment. I think a lot of people with autism do go somewhere to see animals or connect with them in some way, because the few other people I know with autism are all very drawn to animals as well. They don’t question it, but I’d say it’s probably a similar thing to me. It’s an easy situation where you don’t have to worry about anything.

Participants described their ability to learn what their pet communicated through their behaviour, an ability that they found profoundly satisfying and an achievement in and of itself.

I help with cat behaviour and psychology and stuff and help people with some cat issues … Because obviously it’s never the cat, the cat’s just trying to tell them something and they don’t speak cat. So, I just try to find out what the cat’s trying to say and get them to fix it.

Interacting with animals meant being able to drop the mask that many wore in the presence of neurotypicals. Animals were not there to judge them, and the minutiae of communication that often resulted in stressful masking or camouflaging to hide their autistic traits was not an issue when it came to animal relationships. This was empowering as it meant that participants could feel comfortable in who they were:

It is certainly true that with an animal, you don’t need to wear a mask because they don’t know the human social rules that you are breaking. There’s something about the fact that they are not the same species which is helpful. Because an animal is not going ‘doesn’t she know that you have to hold eye contact?’ A cat doesn’t like eye contact. So, they’re not watching you trip over the social rules.

In this sense, animals provided a unique opportunity for participants to gain social contact without the harm that often accompanies traditional human contact. Pets provided company without judgement. They also allowed participants to see themselves as valuable social partners who could make a difference in their lives by taking care of them. Positive interactions with pets allowed participants’ pro-social intentions to shine through without being obscured or distorted by a neurotypical social value system. Participants also learned about and reflected on themselves through the process of caring for animals. As one participant described:

I basically realised that all cats are autistic, and I can’t describe how much I relate to them. They’re very hypersensitive to certain senses and hyposensitive to others. They’re happy to be completely solitary animals but can get very close to specific people that they trust and are very, very loyal to them. They absolutely thrive off routine and don’t like their environment changing. They’re very quiet generally. And also I think most people misunderstand them and don’t communicate well with them because they communicate and show love in a very different way.

### Theme 3. Pets As A Social Lubricant

While participants discussed experiences that left them feeling isolated and unsuccessful, at the same time, animals were a bridge that could connect autistic people to their community. Companion animals provided them with a set script that allowed them to interact with other people more easily: pet-focused conversations fit with autistic preferences for more controlled communication:

It is a very boundaried thing. You chat for five minutes at the most and then you’re away. It’s not demanding. It doesn’t require you to put the mask on, camouflage, pretending you’re neurotypical, saying the right thing. And if it does it’s so brief that it’s not a problem. [When you are with the pet] it’s something to talk about. It’s very clear what you’re supposed to do, the rules are very, very clear. You meet on the walk. You say, ‘Hello Rover, how nice to see you! How are you? Yes, isn’t Sadie beautiful?’

Just as a social script was helpful, being able to use pets as a way to manage feelings of overstimulation was also a key benefit. While feeling socially overwhelmed was common, it was also something that could be difficult to verbalise or explain. Pets could be used as a proxy to manage these types of situations:

I think also it’s quite useful as you’ve got an excuse to sort of bailout. You just say that the dog needs to go outside or you need to take the dog home or whatever, and it’s quite an easy way out…. If it’s too crowded somewhere, it’s an excuse to say ‘The dog wants to go home,’ when really, it’s me.

Experiencing the social world in the company of a pet, which built self-confidence and provided opportunities to engage with other people led to permanent changes for some. Being viewed as more approachable through the company of an animal companion led to more social contact, and by experiencing that social contact, participants felt less anxiety about these types of interactions. They also saw themselves as friendly, caring people through the experience of owning a pet.

Before [adopting Kai], I wouldn’t be the type of person that would go up to a person to strike up a random conversation. Even when it came to a restaurant and I had to get extra napkins or something, I wouldn’t be the person to go ask for it. I’d always get someone else to. But whenever I had my dog with me, I would go out and talk to random people, and I feel like now I’ve improved over time. Now I’m such an outgoing person.

In this sense, pets provided a social outlet in more ways than one. They allowed participants to show their social side and provided a much-needed escape when situations were stressful. Importantly, they provided participants with a social interest and identity they could use to connect with other fellow animal lovers that allowed them a place in the wider community.

### Theme 4. Barriers And Breakthroughs To Pet Ownership

Pet ownership was not something that all participants felt they could handle at this point in their lives. While each had owned pets at some point, about a quarter did not currently own a pet. Those who did also acknowledged that there were sacrifices they had made along the way that made pet ownership possible. All participants acknowledged the barriers that currently existed with regards to making pet ownership possible, particularly for autistic people.

Some of these barriers were financial and included issues with housing and the cost of pet ownership:

It’s not really an option [for me]. It’s not viable. I mean, [my husband and I] live in rented flats, so, we wouldn’t be allowed to have any pets even if I wanted some.

Some participants were worried that aspects of their autistic traits might impede their ability to care for a pet responsibly:

And one thing I have is black and white thinking and I feel like I have that and a lot of indifference. Where I think nothing matters at the moment and I just don’t care about anything. And because of that, I don’t feel like I would be fit to have a pet on a permanent basis.

Despite some of the barriers that came with pet ownership, there were also solutions that participants who were current pet-owners found to compensate for similar issues. For instance, big pets like dogs were challenging to commit to due to the need for constant care and certain types of housing. So participants instead opted for smaller pets like cats, rodents or birds that were more manageable:

My lifestyle doesn’t really fit the dog. So, that’s why I have two cats. Because if I’m away all day when I’m working then they are company for each other. I wouldn’t do it to a dog.

Participants without dogs of their own interacted with the dogs of friends and family, which they used as a proxy for pet ownership a:

I do like seeing my mom’s dogs or my uncles. For example, my uncle has a small dog, which was treated very badly by his past owners. So yes, he is very wary of who he goes to, but I have a connection with him and whenever he sees me he gets really happy. It’s another reason why I don’t really feel like I need to have one of my own because I have my uncle and my mom’s.

However, for some participants, adopting an animal against the odds was itself a breakthrough. It gave them a purpose to take a chance and care for an animal, even if that meant that there would be uncertainty about how they could accommodate their new pet. This need to take care of a pet is what saved some:

The only way I could go on was if I drank loads or did a rap of speed, or you know something else, and, at that point, I didn’t really care about my life. I thought, well you know if I do this and it kills me, then never mind. I didn’t have anything to live for, really. Then once I rescued the cat, and she had all her kittens, that was, you know, that was my reason to live. I thought, you know, I’ve got to stay alive because I’m responsible for her, I’ve got to look after her, I’ve got to look after her babies.

One participant acknowledged that for autistic people, pet ownership could be difficult, but that animal contact is vital. She suggests:

I think that when people choose to get an animal, I think they need to be patient with themselves and learn what is required and then how to do it step by step. It would be awesome if they could have sort of a pet mentor or a pet life coach to show them what to do and to get them onto that schedule.

In this sense, though taking in an animal was not always straightforward and came with, in some cases, new challenges, for many, it was worth it. It gave them the impetus to make changes and take on responsibilities that became a source of pride. That said, there are clearly barriers that come with pet ownership, and these may be magnified in autistic people specifically. Despite this, autistic people came up with solutions that may make animal contact possible (i.e. contact with other people’s pets) and ideas for supporting autistic people if they adopt a pet (i.e. mentorship).

## Discussion study 2

In Study 2, we interviewed autistic people who were current or past pet owners to understand their relationship with pets. Participants reported that owning a pet brought numerous benefits, such as the motivation to go outside and do more exercise. The simple fact that they felt responsible for an animal motivated them to have a more active life. Pets also allowed for calming interactions where the sensory benefits of stroking fur, or being able to vent without expecting a response, improved mental health in times of stress. Autistic people also appreciated pets because human-pet interactions required fewer rules than interactions with neurotypicals. Autistic people did not feel judged by the pets, and they felt they could be themselves without wearing a more socially acceptable mask.

When accompanied by a pet, interactions with neurotypicals also felt safer and more natural because they were brief and guided by simple social rules, and allowed an easy escape if needed. Paired with the confidence of having learnt how to read the behaviour of other living beings, this allowed some participants to develop enough self-confidence to interact with neurotypicals in a variety of settings.

Although all participants acknowledged the benefits pets afforded, participants did not fail to recognise that there were circumstances that could prevent an autistic person from owning a pet. They admitted that it could be too expensive and time-demanding, particularly for large pets. Two participants also expressed that they felt they could not be responsible owners because of their mental state during some periods. Nevertheless, it is important to note that all who were interviewed expressed motivation to overcome these issues, for example, by interacting with family pets or adopting more manageable animals.

## General discussion

Both Study 1 and Study 2 highlight the importance of pets in the lives of autistic people while also revealing some of the unique benefits and challenges that pets provide. Study 1 was in line with previous research (Serpell, 1991, 2000) in showing that pet owners (autistic and NT alike) had higher satisfaction with life than non-pet owners, regardless of pet type. This was also confirmed in the interviews with autistic individuals indicating that owning a pet increases the motivation to have an active life, and communicating both with pets, and through pets, provided a mechanism for managing stressful situations when they arose.

Furthermore, Study 1 indicates that autistic people who owned pets scored similarly to NTs on measures relating to pet-focused social attitudes, including attachment, ethics and anthropomorphism. These findingssupport the claim that human-animal bonds are intact in autistic populations (Carlisle, 2015; Grandin et al., 2015; Prothmann et al., 2009). It also suggests that theories positing reduced social motivation in autistic populations are not compatible with the intact interest and motivation autistic people display towards non-human social creatures (Atherton & Cross, 2018; Valiyamattam et al., 2020). Indeed, Study 1 showed that the level of attachment between the autistic pet owner and their pet were positively correlated with mental health variables including social anxiety, loneliness and percieved social support, and this was not significantly correlated in NTs. As indicated by interviews in Study 2, pet owners opened up to their pets when they were vulnerable, went out of their comfort zones to fulfill their pets' social or physical needs, and spent time engaging with their pets on a deeper level. All of these behaviors are indicative of a secure attachment and increased prosociality, and cultivating this in human-animal dyads may be particularly critical for autistic people. While autistic people are capable of forming secure attachments in line with NTs, there are risk factors that may impede attachment early in life (Teague et al., 2017), and friendships later in life (Sedgewick et al., 2019). Having a secure attachment to an animal companion may be an important addition to autistic people's lives, as it may be provide protective mental health benefits.      

In line with improved mental health in relation to pet attachment, autistic people were more also likely to substitute their pets for people, meaning that their pets were serving the purpose of human contact. We found that what drove people substitution in our sample was social avoidance, a well-documented facet of autism that corresponds to the social anxiety and negative social experiences many on the spectrum experience (Bejerot et al., 2014; Cappadocia et al., 2012; Eriksson et al., 2013). In other words, social avoidance motivated autistic individuals to fill this need for human contact with a pet substitute. However, the interviews indicated that pets did not take away from participants’ engagement with other people, and instead enhanced their sense of self and gave them a pathway to develop social skills, supporting previous claims (Serpell, 2000). Investigating the social gains and mental health of autistic adults before and after caring for a pet would be an important area of work towards understanding the transfer between the pet-human bond and the human-human bond, and the benefits of daily animal companionship.

Despite these benefits, we are not suggesting that autistic people should automatically adopt pets. As described in Study 2 and highlighted in Study 1’s finding that pet ownership is less common, there are clear barriers for autistic people to owning a pet. Some could not currently own pets due to housing restrictions, irregular income and fear that aspects of their condition would impede their duty of care. Others who were pet owners also attested to these challenges. Yet, they also felt that having a pet forced them to overcome these challenges, be it through illegally housing a pet, purchasing a smaller pet, or continually being aware of their responsibilities to their pet, which motivated them to keep well.

Finding ways to overcome these barriers may therefore be necessary for the wellbeing of the autistic population. One possibility suggested by several participants in Study 2 would be to develop a community program in which autistic people can be mentored on pet ownership and perhaps financially supported in the adoption of animals in shelters. Such a program would provide autistic adults with the opportunity to own a pet while also assisting them with animal care costs. Furthermore, a pet mentor could monitor the wellbeing of the owner and pet and give guidance on how to care for the animal.

Another possibility could be to create social groups centred around pet ownership, including meetups and communication channels, which would provide opportunities for the kind of social contact that participants in Study 2 cited as being particularly comfortable. There is also a high need for volunteers at animal shelters, where research suggests that animal contact is particularly beneficial to animals housed in such facilities (Coppola et al., 2006). This, too, could be an essential way for autistic people to gain both human and animal contact, and help animals in need. It could also lead to employment opportunities for the autistic community, who are in particular need of occupational environments that fit their unique skills and interests (Harmuth et al., 2018).  

In conclusion, autistic adult populations are more susceptible to mental health issues that reduce wellbeing (Atherton et al., 2021), which animal contact may improve (McConnell et al., 2019). Our study shows that pet ownershipis related to higher quality of life in all people, but pet attachment is specifically related to better mental health in autistic adults. Furthermore, in autistic adults alone having a pet can provide specific social benefits that may combat loneliness and fulfill social needs traditionally attained through human interactions. Pets are important to autistic adults not only for the benefits their compationship provides in and of itself, but also through the ways animals bring their owners into the community and provide them with a positive social identity. Autistic adults are perhaps the most in need of timely, cost-effective interventions that can be undertaken with some amount of independence, as they often report being overlooked in current healthcare systems (Camm-Crosbie et al., 2019). Indeed, research suggests that the resources allotted to autistic adults are meagre compared to those allocated to children (Gerhardt & Lainer, 2011), with an estimated 96% of children receiving formal intervention in the United States (Monz et al., 2019). Autistic adults, in contrast, are much more likely to live outside of these traditional systems of care, and are often competing for time and resources with other special needs groups (Crane et al., 2019; Jones et al., 2014).

Our results suggest that animal-based approaches to improving mental health outcomes in autistic adults would be a particularly effective intervention. Animal contact in the form of pet ownership could be a form of social support for autistic adults that is relatively cost-effective from a public funding perspective. Importantly, establishing an animal-human bond between an autistic person and a pet may provide a unique benefit over and above other interventions typically offered. As autistic people often experience social rejection, criticism, and even bullying when interacting socially with other humans, the positive social interactions they experience when caring for an animal appears to be critical in boosting social self-efficacy and fufilling social needs. As many of the same processes are implicated in animal social cognition and human social cognition, gaining efficacy in understanding an animal’s wants and needs could be a valuable way to build skills that may transfer to other settings. Importantly, attachment through animal contact is naturalistic and based on genuine relationships rather than scripted interactions in artificial settings. Future research may want to investigate how to harness the benefits of human-animal contact further to improve the lives of autistic people.

## Supplementary Materials

**Table A1:** Kolmogorov-Smirnov (KS) normality tests

*Cluster*	*Variable*	*KS test*
Pet Relationship	General Attachment	KS(611) = 0.16***
	People Substitution	KS(611) = 0.12***
	Animal Rights	KS(611) = 0.19***
	Pet Rating Social	KS(611) = 0.05**
	Pet Rating Non-Social	KS(611) = 0.04**
Quality of Life	Performance Anxiety	KS(611) = 0.05***
	Social Anxiety	KS(611) = 0.06***
	Performance Avoidance	KS(611) = 0.07***
	Social Avoidance	KS(611) = 0.07***
	Life Satisfaction	KS(611) = 0.07***
	Loneliness	KS(611) = 0.05**

*p < .05, **p < .01, ***p < .001

KS: Kolmogorov-Smirnov test

**Table A2:** Spearman’s correlations within the pet relationship cluster

	*People Substitution*	*Animal Rights*	*Pet Rating Social*	*Pet Rating Non-Social*
*General Attachment*	0.75***	0.74***	0.47***	0.18***
*People Substitution*		0.65***	0.39***	0.20***
*Animal Rights*			0.31***	0.11**
*Pet Rating Social*				0.28***

*p < .05, **p < .01, ***p < .001
